# Efficacy of ^177^Lu-PSMA-617 Therapy in mCRPC Patients with Liver Metastases: Insights into Survival Outcomes and Predictors of Response

**DOI:** 10.3390/biomedicines13030569

**Published:** 2025-02-24

**Authors:** Ebuzer Kalender, Edanur Ekinci, Umut Elboğa, Ertan Şahin

**Affiliations:** Department of Nuclear Medicine, School of Medicine, Gaziantep University, Gaziantep 27310, Turkey; eda.nur.eknci@hotmail.com (E.E.); umutelboga@hotmail.com (U.E.); er_ahin@yahoo.com (E.Ş.)

**Keywords:** metastatic castration-resistant prostate cancer, ^177^Lu-PSMA-617, radioligand therapy, liver metastases, progression-free survival, overall survival, PSA, SUVmax

## Abstract

**Objectives:** Metastatic castration-resistant prostate cancer (mCRPC) is associated with poor prognosis, particularly in cases of liver metastases. ^177^Lu-PSMA-617 (commercially known as Pluvicto) is an FDA-approved radioligand therapy for mCRPC patients. This study aimed to evaluate the efficacy of ^177^Lu-PSMA-617 radioligand therapy (RLT) in mCRPC patients with liver metastases, focusing on progression-free survival (PFS), overall survival (OS), and factors influencing treatment response. **Materials and Methods:** This retrospective study included mCRPC patients (n = 32) with liver metastases treated with Lu-PSMA-617. Patient data, including prostate-specific antigen (PSA) levels, liver SUVmax values, Lutetium-PSMA therapy cycles, and survival outcomes, were collected. Kaplan–Meier survival analysis was used to calculate PFS and OS, while regression analysis was employed to identify factors associated with treatment response. **Results:** The median PFS and OS were 6 and 9 months, respectively. Partial regression was observed in patients with significantly lower PSA levels (median: 90.0 ng/mL, range: 22–699 ng/mL, *p* = 0.001) and liver SUVmax values (median: 17.9, range: 8.3–57.0, *p* = 0.008). A higher number of Lutetium-PSMA cycles correlated with improved treatment response (*p* = 0.010) and reduced liver SUVmax values (*p* = 0.043). **Conclusions:** Lu-PSMA-617 therapy is effective in managing mCRPC with liver metastases. Increased intensity of therapy exposure, reflected by a higher number of treatment cycles, is associated with a greater biochemical response, as indicated by reduced PSA levels, thereby supporting the rationale for personalized treatment strategies. These findings support the use of Lu-PSMA-617 in mCRPC patients with liver metastases, warranting further prospective studies.

## 1. Introduction

Prostate cancer is the second most common type of cancer in men worldwide and has a significant share in cancer-related deaths [1]. Although prostate cancer is generally a slowly progressing disease, it metastasizes in advanced stages, reducing quality of life and increasing mortality rates. Metastatic castration-resistant prostate cancer (mCRPC) is a form that does not respond to hormone therapy, and advanced treatment methods such as chemotherapy and radioligand therapy are required in these patients [2,3].

Autopsy series reveal that prostate cancer metastases are most frequently seen in the bone, lung, and liver. Liver metastases, in particular, are detected in approximately 8.6% of prostate cancer patients, and this rate negatively affects the course of the disease [4]. Liver metastases are the second most common site of visceral metastases in mCRPC patients, and this situation is associated with poor prognosis [4].

Prostate cancer is stratified into risk categories based on factors such as PSA levels, Gleason score, and tumor stage. These categories—very low, low, intermediate, high, and very high risk—aid in guiding treatment decisions. In metastatic prostate cancer, various therapeutic options are considered. While immune checkpoint inhibitors targeting the PD-1/PD-L1 pathway have shown promise in several cancers, their efficacy in prostate cancer has been limited. Studies indicate that PD-L1 inhibitors, such as pembrolizumab and avelumab, have demonstrated limited clinical efficacy in men with metastatic prostate cancer [5,6,7].

Prostate-specific membrane antigen (PSMA) is a transmembrane glycoprotein that is highly expressed in prostate cancer cells, particularly in advanced and metastatic stages of the disease, and its expression levels increase as tumor aggressiveness escalates [8]. PSMA is virtually absent in most normal tissues, except for low-level expression in the salivary glands, kidneys, and small intestine, making it a highly specific target for prostate cancer imaging and therapy, as well as an ideal biomarker for radiolabeled treatments [9,10,11]. ^177^Lu-PSMA-617 (Lu-PSMA-617) radiolabeled radioligand therapy (RLT) binds to PSMA-expressing tumor cells, delivering targeted radiation and leaving minimal damage to healthy tissues [11,12,13,14,15].

Lu-PSMA-617 was approved by the US Food and Drug Administration (FDA) in March 2022 for use in mCRPC patients, offering a new treatment option for patients resistant to chemotherapy and unresponsive to antihormonal therapy [16]. Clinical studies have shown that Lu-PSMA-617 reduces progression by 60% and overall mortality by 38% when combined with standard therapy [13,16,17,18]. Lu-PSMA-617, used in the treatment of metastatic castration-resistant prostate cancer (mCRPC), has shown promising results in clinical studies. In particular, the VISION study reported that this treatment improved overall survival and extended radiographic progression-free survival when used in combination with standard care [13]. However, there are limited studies on the therapeutic efficacy of Lu-PSMA-617 on liver metastases [14]. Current studies focusing on prostate cancer metastases mostly focus on bone metastases [14]. Liver metastases can have more serious effects on the survival of patients and therefore the treatment of these metastases is of great importance.

The aim of this study was to retrospectively evaluate the efficacy of Lu-PSMA-617 treatment for liver metastases in mCRPC patients and to examine the relationship between liver lesion response and median survival. This retrospective study will provide important data to analyze the response to treatment and optimize the use of Lu-PSMA-617 in future clinical applications.

## 2. Materials and Methods

### 2.1. Study Design and Study Population

This study was approved by the Non-Interventional Clinical Research Ethics Committee of Gaziantep University (Approval Date: 4 December 2024; Approval Number: 2024/442). It was conducted in compliance with the principles of the Declaration of Helsinki. The study employed a retrospective design, including patients who underwent Lu-PSMA-617 therapy and had liver metastases due to mCRPC between 1 January 2020, and 1 October 2024, at the Department of Nuclear Medicine, Faculty of Medicine, Gaziantep University. ^177^Lu-PSMA-617 (commercially known as Pluvicto) is an FDA-approved radioligand therapy for mCRPC patients.

Inclusion Criteria:

1. Patients with a histopathologically confirmed diagnosis of prostate cancer.
2. Patients who received Lu-PSMA-617 therapy and were identified to have liver metastases.
3. Patients aged 18 years and older.

Exclusion Criteria:

1. Patients without a histopathological diagnosis of prostate cancer.
2. Patients younger than 18 years of age.

Patient data were retrospectively collected from the electronic health records (EHR) system of Gaziantep University Şahinbey Research and Training Hospital. While all patients had a confirmed diagnosis of prostate cancer, comorbidities such as hypertension, diabetes, or cardiovascular diseases were not systematically assessed due to the retrospective nature of the study. Routine follow-up findings, including Ga-68 PSMA PET/CT imaging and laboratory results, were analyzed. In this study, parameters such as prostate-specific antigen (PSA) levels, Gleason scores, liver SUVmax values, number of Lutetium-PSMA cycles, Eastern Cooperative Oncology Group (ECOG) performance status, response rates of liver metastases, progression status, and survival outcomes were evaluated. Ga-68 PSMA PET/CT was the primary imaging modality used for assessing liver metastases in this study. Additional imaging techniques such as contrast-enhanced CT or MRI were not routinely performed, as Ga-68 PSMA PET/CT has demonstrated superior sensitivity and specificity for detecting PSMA-expressing metastatic lesions. PET/CT also provides direct functional and molecular imaging relevant to Lu-PSMA-617 therapy, making it the preferred modality for staging and response assessment in mCRPC patients. Response to therapy was assessed based on PSA dynamics, liver SUVmax values, and imaging findings. Patients demonstrating a reduction in PSA and SUVmax values or a decrease in tumor burden were classified as ‘Yes’ while those with increasing values or disease progression were considered ‘No’.

SUVmax values were calculated using AW VolumeShare v.2. PET/CT software (GE Healthcare, Milwaukee, WI, USA), with volumes of interest (VOIs) manually drawn around liver metastases. Tumor response was assessed based on PSMA PET/CT criteria and RECIST 1.1 guidelines, with all analyses manually reviewed by nuclear medicine specialists to ensure accuracy. Ga-68 PSMA PET/CT was used for baseline and post-treatment evaluations, providing a prognostic marker for tumor burden. Higher baseline SUVmax values indicated increased disease severity, while post-treatment reductions reflected therapeutic response and were associated with improved survival outcomes. This quantitative assessment helped monitor treatment efficacy and guide clinical decisions.

Ga-68 PSMA PET/CT was used as the primary imaging modality for metastatic castration-resistant prostate cancer (mCRPC) in this study, as it provides high specificity for PSMA-expressing tumors. FDG PET/CT was not routinely performed, as PSMA PET/CT has been shown to be more effective in detecting prostate cancer metastases. FDG PET/CT is typically reserved for cases with suspected dedifferentiation or neuroendocrine differentiation, which were not the primary focus of this study.

### 2.2. Preparation, Calibration, and Dosimetry of Radioligand Therapy

The ^177^Lu-PSMA-617 radioligand therapy used in this study was commercially obtained from Novartis (Pluvicto™, lutetium Lu 177 vipivotide tetraxetan, Novartis, Indianapolis, IN, USA) and was administered according to the manufacturer’s specifications. The radiopharmaceutical was delivered in pre-calibrated, sterile, ready-to-use vials, ensuring high purity and consistency across treatment cycles. The administered activity per cycle ranged from 5.5 to 7.4 GBq, based on patient-specific parameters, including renal function and tumor burden. Dosimetry calculations were performed following standard clinical protocols. Post-therapy SPECT/CT imaging was conducted to assess the biodistribution and radiation dose to organs at risk, particularly the kidneys, bone marrow, and salivary glands. The absorbed dose estimations were based on the Medical Internal Radiation Dose (MIRD) schema, and therapy cycles were adjusted accordingly to optimize efficacy while minimizing toxicity. Unlike in-house synthesized ^177^Lu-PSMA-617, which requires local radiolabeling and stringent quality control measures, Pluvicto is produced under Good Manufacturing Practice (GMP) regulations by Novartis. This ensures batch-to-batch consistency, high radiochemical purity, and standardized pharmacokinetics, making it a widely accepted option in clinical practice. Both formulations share the same mechanism of action, targeting PSMA-expressing prostate cancer cells and delivering beta-radiation therapy to metastatic sites.

### 2.3. Statistical Analysis

Statistical analyses were performed using SPSS software version 27.0 (IBM Corp., Armonk, NY, USA). Descriptive statistics were presented as mean ± standard deviation (SD) or median (minimum–maximum) for continuous variables, and as frequencies and percentages for categorical variables. The Wilcoxon Signed-Rank Test was used to evaluate differences between pre-treatment and post-treatment PSA and SUVmax values. The Mann–Whitney U Test was applied to compare groups based on progression status. The relationship between Lutetium PSMA cycle number and clinical parameters was assessed using Spearman correlation analysis. To identify factors affecting response rates of liver metastases (regression), linear regression analysis was performed. The chi-square test was used to evaluate the association between progression status and mortality outcomes. The Kaplan–Meier method was used to calculate the median progression-free survival (PFS) and overall survival (OS). PFS was defined as the time from treatment initiation to disease progression or last follow-up, while OS was defined as the time from treatment initiation to death or last follow-up. Kaplan–Meier survival curves were generated to illustrate survival probabilities, and comparisons between groups were conducted using the log-rank test. A *p*-value <0.05 was considered statistically significant.

## 3. Results

Sociodemographic data and other parameters are shown in Table 1. The study included 32 patients with a median age of 72 years (range: 57–90 years). The median follow-up period was 6 months (range: 3–15 months). The Gleason score of the patients ranged from 6 to 10, with a median score of 9. Patients received a median of 4 cycles of Lu-PSMA therapy (range: 2–7 cycles). Regarding prior treatments, 15.6% of the patients had received abiraterone, 59.4% bicalutamide, 53.1% docetaxel, 59.4% enzalutamide (Enza), 68.8% goserelin, and 53.1% leuprolide. Additionally, metastatic involvement outside the liver was observed in 71.9% of patients in both lymph nodes and bones, while bone-only metastases were detected in 25% of patients, and lymph node-only metastases were present in 3.1% of patients.

In terms of liver metastases response rates, numerical progression was observed in 25%, molecular progression in 15.6%, and both numerical and molecular progression in 21.9% of patients. Numerical regression was seen in 3.1%, molecular regression in 15.6%, and combined numerical and molecular regression in 18.8% of patients. The ECOG performance status was 0 in 15.6% of patients, 1 in 56.3%, and 2 in 28.1%. Progression status showed that 62.5% of patients experienced disease progression, while 37.5% had partial regression. Survival analysis revealed that 87.5% of patients were alive at the end of the follow-up period, while 12.5% had died (Table 1).

The comparison of pre-treatment and post-treatment parameters showed a reduction in PSA levels and liver SUVmax values following therapy. The median PSA level decreased from 78.0 ng/mL (range: 5.0–602.0 ng/mL) before treatment to 70.5 ng/mL (range: 5.0–699.0 ng/mL) after treatment; however, this difference was not statistically significant (*p* = 0.121). Similarly, the liver SUVmax values declined from a median of 16.75 (range: 7.2–42.0) before treatment to 15.4 (range: 8.3–57.0) after treatment, without reaching statistical significance (*p* = 0.242). These findings indicate that while there was a downward trend in these parameters post-treatment, the changes were not significant within the observed cohort (Table 2).

**Figure 1:**
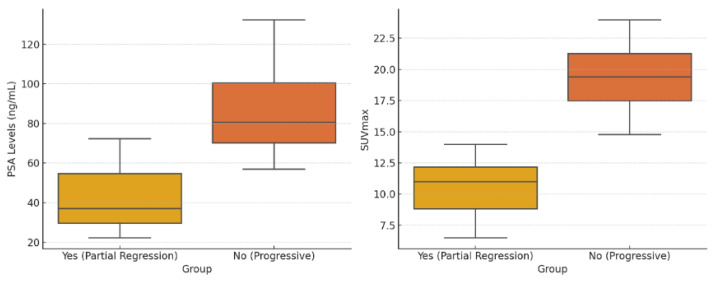
Comparison of the PSA levels and SUVmax in Yes (Partial Regression) vs. No (Progressive) Groups.

Comparison of parameters according to progression status in patients receiving Lu-PSMA-617 treatment was shown in Table 3. The comparison of parameters based on progression status in patients treated with Lu-PSMA-617 revealed significant differences between the progressive and partial regressive groups. The median follow-up period was significantly shorter in the progressive group (5 months, range: 3–13 months) compared to the partial regressive group (8 months, range: 4–15 months) (*p* = 0.009). Similarly, the number of Lutetium-PSMA cycles was lower in the progressive group (median: 3, range: 2–7) compared to the partial regressive group (median: 5, range: 3–7) (*p* = 0.003). PSA levels were significantly higher in the progressive group (median: 90.0 ng/mL, range: 22–699 ng/mL) compared to the partial regressive group (median: 44.5 ng/mL, range: 5–563 ng/mL) (*p* = 0.001). Furthermore, the liver SUVmax values were also significantly higher in the progressive group (median: 17.9, range: 8.3–57.0) compared to the partial regressive group (median: 11.8, range: 8.7–39.8) (*p* = 0.008) (Table 3, Figure 1).

**Figure 2:**
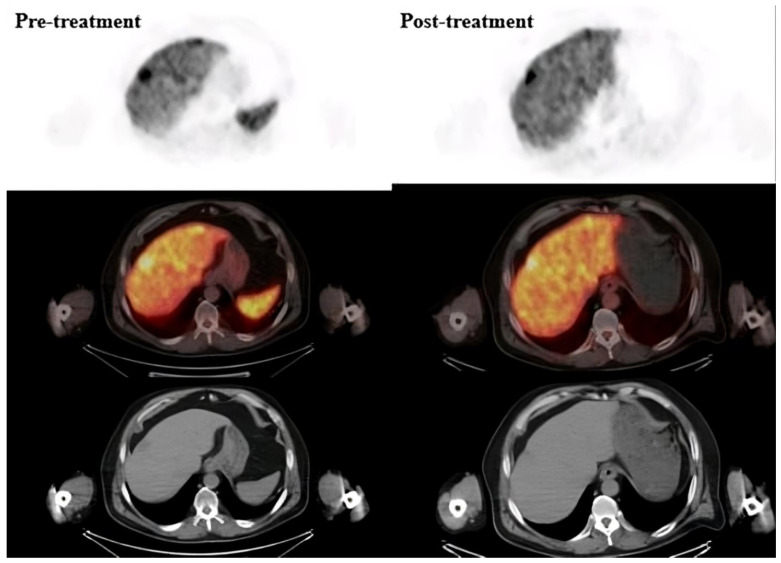
The pre-and post-treatment Ga-68 PSMA PET/CT images of a representative patient. The pre-treatment image shows liver lesions with high PSMA uptake (SUVmax: 60–68), indicating significant metastatic involvement. The post-treatment image, obtained after two cycles of Lu-PSMA-617 therapy, demonstrates a reduction in both lesion size and molecular activity, with a corresponding decrease in SUVmax values (SUVmax: 30–35).

Regarding the response rates of liver metastases, numerical, molecular, or combined numerical and molecular progression was observed exclusively in the progressive group, while partial regression was predominantly associated with molecular or combined molecular and numerical regression in the partial regressive group (*p* < 0.001). The ECOG performance scores also differed between groups, with worse performance scores more commonly observed in the progressive group (*p* = 0.05). Survival analysis revealed that 20% of patients in the progressive group had died, whereas no deaths were reported in the partial regressive group, although this difference did not reach statistical significance (*p* = 0.103). These findings indicate that better treatment response and lower disease progression are associated with higher Lutetium-PSMA cycles, lower PSA levels, and lower liver SUVmax values (Table 3, Figure 2).

**Figure 3:**
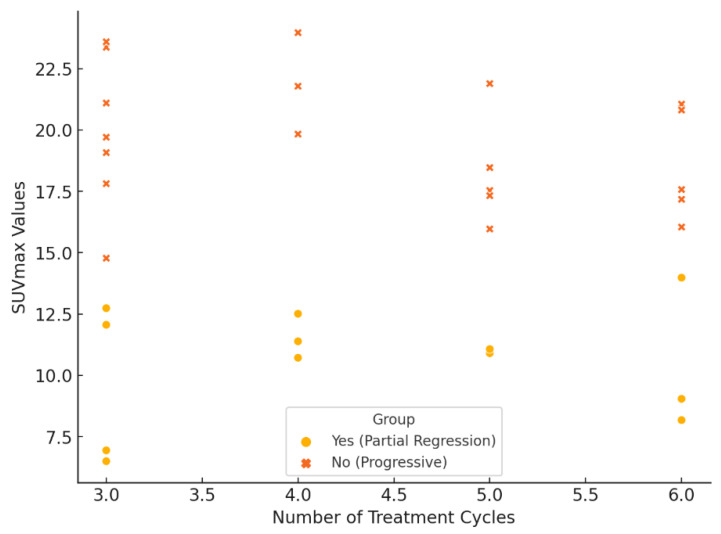
Correlation between Treatment Cycles and SUVmax Reduction.

Response rates of liver metastases (Regression) and linear regression analysis of parameters was shown in Table 4. The correlation analysis between Lutetium-PSMA cycle number and various clinical parameters demonstrated significant associations with certain variables. While no significant correlation was observed between the cycle number and Gleason score (r = 0.024, *p* = 0.898), a moderate positive correlation was identified between the number of cycles and PSA levels (r = 0.305, *p* = 0.089), though it did not reach statistical significance. A significant negative correlation was found between the number of cycles and liver SUVmax values (r = −0.359, *p* = 0.043), indicating that higher cycle numbers were associated with lower liver SUVmax values. Furthermore, a significant positive correlation was observed between the number of cycles and response rates of liver metastases (progression to regression) (r = 0.450, *p* = 0.010), suggesting that an increased number of cycles is linked to improved treatment response in liver metastases. These findings highlight the impact of the number of Lutetium-PSMA therapy cycles on treatment efficacy, particularly in reducing liver SUVmax values and enhancing response rates of liver metastases (Table 4, Figure 3).

Response rates of liver metastases (Regression) and linear regression analysis of parameters were shown in Table 5. The linear regression analysis identified several factors significantly associated with the response rates of liver metastases in patients treated with Lu-PSMA-617. The number of Lutetium-PSMA therapy cycles was a significant predictor, with a regression coefficient (B) of 0.939 (95% CI: 0.860–1.182, *p* = 0.027), indicating that an increased number of therapy cycles positively impacted the response to treatment. Similarly, liver SUVmax values were positively correlated with response rates, with a regression coefficient of 0.180 (95% CI: 0.002–0.045, *p* = 0.037), suggesting that lower SUVmax values were associated with improved response to therapy. Additionally, progression status (partial regression) showed a strong association with response rates, with a regression coefficient of 3.945 (95% CI: 3.189–4.702, *p* < 0.001), emphasizing the significant role of disease regression in predicting treatment outcomes. These findings highlight the importance of optimizing treatment cycles and monitoring liver SUVmax values to enhance the efficacy of Lu-PSMA-617 therapy in patients with liver metastases (Table 5).

**Figure 4:**
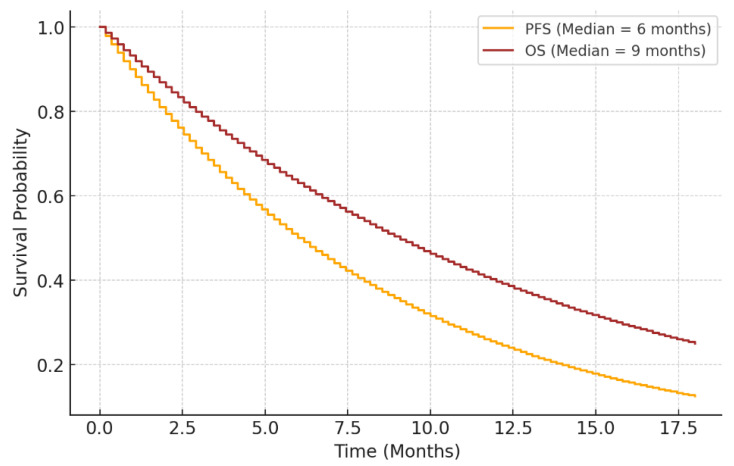
Kaplan–Meier Survival Curve for PFS and OS.

The median PFS was calculated as 6 months, indicating the duration during which patients-maintained disease stability or partial regression following Lu-PSMA-617 therapy. Additionally, the median OS was determined to be 9 months, reflecting the survival time from the initiation of treatment to death or the end of follow-up. These findings emphasize the therapeutic potential of Lu-PSMA-617 in managing mCRPC with liver metastases. The observed PFS and OS outcomes align with previous studies in the literature, highlighting the clinical efficacy of this targeted radioligand therapy in improving disease control and survival in this challenging patient population (Figure 4).

## 4. Discussion

This study provided an important contribution to the limited number of studies evaluating the efficacy of Lu-PSMA-617 treatment in patients with mCRPC and liver metastases. While the literature generally focuses on bone metastases, our study provides specific and detailed data for this patient group by examining the effects on liver metastases in depth. Our findings, which show that progression and partial regression are strongly associated with survival and that the number of treatment cycles is a critical factor that increases treatment efficacy, are important guides for the development of personalized treatment plans. In addition, we analyze the treatment response with biochemical and imaging parameters such as PSA and SUVmax, emphasizing the importance of these criteria in prognostication and follow-up.

In our study, the observed decrease in PSA levels from 78.0 to 70.5 was not statistically significant. This finding aligns with existing literature indicating that patients with liver metastases often exhibit a less pronounced PSA response to Lu-PSMA-617 therapy. For instance, Muniz et al. reported that the presence of liver metastasis predicts poorer outcomes in patients receiving Lu-PSMA-617 treatment, with a lower rate of PSA decline compared to patients without liver involvement [19]. Similarly, Seifert et al. found that while Lu-PSMA-617 therapy frequently controls liver metastases, the hepatic tumor burden did not appear to influence treatment efficacy, suggesting that Lu-PSMA-617 is effective even in patients with substantial liver involvement [15]. These findings suggest that the limited PSA response observed in our cohort may be attributed to the presence of liver metastases, which are associated with a more aggressive disease phenotype and a microenvironment less responsive to PSMA-targeted therapies. Further research is warranted to explore combination treatment strategies or alternative therapies to enhance efficacy in this subset of patients.

Liver metastases, though less prevalent than bone metastases in prostate cancer, are linked to a poorer prognosis and more aggressive disease progression [20]. Rahbar et al. conducted a German multicenter study evaluating the efficacy and safety of Lu-PSMA-617 radioligand therapy in patients with mCRPC. The study reported that 45% of patients experienced a PSA decline of ≥50%, indicating a significant biochemical response. Additionally, the treatment was well-tolerated, with manageable adverse effects [21]. Khreish et al. conducted a pivotal study evaluating the efficacy of Lu-PSMA-617 therapy in patients with mCRPC and liver metastases. Their findings demonstrated that hepatic disease control, defined as either complete or partial response, was achieved in 46% of patients, with a median progression-free survival (PFS) of 5.7 months and a median overall survival (OS) of 11.7 months. Importantly, patients who achieved hepatic disease control had significantly longer survival compared to those with progressive liver disease. Additionally, a PSA decline of ≥50% after two therapy cycles and a good baseline performance status (ECOG 0–1) were independently associated with improved survival outcomes. These results underscore the potential of Lu-PSMA-617 therapy in effectively managing liver metastases and improving clinical outcomes, consistent with our study findings that highlight the importance of optimizing therapy cycles and achieving partial regression for better survival in mCRPC patients [20]. In our study, patients with liver metastases exhibited varied responses to Lu-PSMA-617 therapy. Consistent with prior research, we observed that patients achieving partial regression had significantly lower PSA levels and liver SUVmax values compared to those with progressive disease. Previous studies have also demonstrated that a decrease in PSA levels correlates with improved clinical outcomes in mCRPC patients [13].

Chehade et al. discuss the correlation between PSA dynamics and outcomes in patients undergoing Lu-PSMA-617 treatment for mCRPC. They emphasize the importance of refining predictive and prognostic biomarkers to enhance patient selection and treatment efficacy. The authors highlight that PSA responses can serve as valuable indicators of therapeutic benefit, aiding in the optimization of personalized treatment strategies for mCRPC patients [22]. The VISION trial demonstrated that adding ^177^Lu-PSMA-617 to standard care significantly improved overall survival and radiographic progression-free survival in patients with mCRPC. However, it is important to note that the trial primarily included patients with bone metastases, and the presence of liver metastases was associated with poorer outcomes. Specifically, patients with liver involvement had a higher risk of disease progression and reduced overall survival compared to those without liver metastases. This finding aligns with other studies indicating that liver metastases in mCRPC are associated with a more aggressive disease course and poorer prognosis. For instance, a study by Gafita et al. reported that the presence of liver metastasis predicts poorer outcomes in patients receiving ^177^Lu-PSMA-617 treatment [19]. These observations underscore the need for tailored therapeutic strategies and further research to improve outcomes for mCRPC patients with liver involvement.

A notable observation in our study was the significant correlation between the number of Lutetium-PSMA cycles and liver SUVmax values. Patients receiving a higher number of therapy cycles demonstrated lower SUVmax values, indicating reduced metabolic activity in liver lesions. Calais et al. conducted a prospective multicenter phase 2 trial (RESIST-PC) evaluating the safety of ^177^Lu-PSMA-617 radioligand therapy in patients with mCRPC. The study reported that the treatment was well-tolerated, with manageable adverse effects, and demonstrated promising efficacy, including significant reductions in PSA levels. These findings align with our results, where patients undergoing Lu-PSMA-617 therapy exhibited favorable safety profiles and notable decreases in PSA levels, underscoring the potential of this therapy in managing mCRPC [23]. Khreish et al. conducted a prospective study involving 254 patients with mCRPC undergoing Lu-PSMA-617 RLT [12]. The study reported a prostate-specific antigen (PSA) decline of ≥50% in 43% of patients, indicating a significant biochemical response. The median OS was 14 months, with patients exhibiting a PSA decline of ≥50% experiencing a notably longer OS compared to those with less pronounced PSA reductions [12]. Violet et al. conducted a single-center phase II prospective trial involving 50 patients with mCRPC treated with Lu-PSMA-617 theragnostic. The study reported a prostate-specific antigen (PSA) decline of ≥50% in 64% of patients, indicating a significant biochemical response. The median OS was 13.3 months, and the treatment was well-tolerated with manageable adverse effects [24]. These findings align with our results, where patients achieving partial regression demonstrated significantly lower PSA levels and improved survival outcomes, underscoring the prognostic value of PSA response in mCRPC treatment. Furthermore, regression analysis in our study identified the number of therapy cycles as a key predictor of treatment response, reinforcing the importance of personalized treatment protocols to maximize therapeutic benefits.

Our study also observed that progression status was a strong determinant of survival outcomes. Patients with partial regression exhibited better survival compared to those with progressive disease. Sartor et al. conducted the VISION trial, a phase 3 study evaluating the efficacy of Lu-PSMA-617 in patients with mCRPC. The trial demonstrated that patients receiving Lu-PSMA-617 in addition to standard care had a significant improvement in overall survival compared to those receiving standard care alone. Additionally, the treatment group exhibited a higher rate of progression-free survival. These findings align with our study, where patients undergoing Lu-PSMA-617 therapy showed improved survival outcomes, underscoring the therapeutic potential of this targeted radioligand therapy in mCRPC management [14]. SBRT (Stereotactic Body Radiotherapy) has been explored as a potential salvage therapy for liver metastases in oligometastatic prostate cancer, offering high-dose targeted radiation with favorable local control rates. However, unlike systemic Lu-PSMA-617 therapy, SBRT is a localized treatment and is more suitable for patients with limited metastatic disease. Future studies may explore the combined use of SBRT and Lu-PSMA-617, particularly in cases of residual or progressing liver metastases post-radioligand therapy [25,26,27].

The PSMAfore trial demonstrated that ^177^Lu-PSMA-617 significantly prolonged progression-free survival in chemotherapy-naïve mCRPC patients compared to ARPIs alone. While this trial focused on earlier-stage mCRPC, our study specifically evaluated ^177^Lu-PSMA-617 in patients with liver metastases, a subgroup with a generally poorer prognosis [28]. Our findings suggest that SUVmax reduction in liver metastases may serve as an additional prognostic indicator for treatment response, complementing existing data from PSMAfore and other trials.

Seifert et al. conducted a German multicenter study evaluating the safety and efficacy of extended Lu-PSMA-617 therapy in patients with mCRPC. The study demonstrated that extended cycles of Lu-PSMA are both safe and effective, with patients receiving additional treatment cycles showing improved PFS and OS [15]. These findings align with our results, where a higher number of Lu-PSMA-617therapy cycles correlated with reduced metabolic activity in liver lesions and better survival outcomes, underscoring the importance of personalized treatment protocols to maximize therapeutic benefits in mCRPC patients.

### Limitations of the Study

This study has several limitations that should be acknowledged. The present study provides valuable insights into the efficacy of Lu-PSMA-617 therapy for liver metastases in mCRPC; however, several limitations must be acknowledged. First, the retrospective nature of the study introduces potential biases, including selection bias and incomplete data collection, which could limit the generalizability of the findings. Additionally, the relatively small sample size of 32 patients reduces the statistical power to detect subtle differences or associations and may not represent the broader mCRPC patient population. The median follow-up period of six months is relatively short, limiting the ability to assess long-term survival benefits, durability of response, and late toxicities. This limitation is partly due to the retrospective design and the inclusion of patients with aggressive disease and poor prognosis, which may contribute to shorter follow-up availability. One notable limitation is the reliance on SUVmax and PSA levels as primary markers of treatment response. While SUVmax reduction reflects decreased PSMA expression and metabolic activity, heterogeneous PSMA expression within tumors and the influence of non-tumor factors on SUVmax values may lead to variability in response assessment. Similarly, PSA response is not always indicative of treatment efficacy, particularly in patients with neuroendocrine differentiation or discordant imaging findings. Additional imaging modalities, such as contrast-enhanced MRI or diffusion-weighted imaging (DWI), may provide complementary information on tumor viability and perfusion changes following treatment. Moreover, emerging blood-based biomarkers, including circulating tumor DNA (ctDNA) and exosomal PSMA levels, could offer more precise insights into tumor response and resistance mechanisms. Future studies should explore multimodal assessment strategies to enhance the accuracy of treatment response evaluation in mCRPC patients receiving Lu-PSMA-617 therapy. While our study primarily focused on the impact of Lu-PSMA-617 therapy in mCRPC patients with liver metastases, we acknowledge that comorbid conditions may have influenced survival outcomes. Additionally, as cause-of-death analysis was not systematically performed, we cannot exclude the potential contribution of non-cancer-related factors to mortality. Future prospective studies with longer follow-up durations are warranted to better evaluate long-term treatment outcomes and late adverse effects of Lu-PSMA-617 therapy in patients with liver metastases.

## 5. Conclusions

In conclusion, this study demonstrates that Lu-PSMA-617 therapy is an effective treatment modality for liver metastases in mCRPC patients. Higher treatment cycles, lower PSA levels, and reduced liver SUVmax values are associated with better treatment responses. These findings underscore the need for personalized treatment approaches to optimize therapy cycles and monitor key biomarkers for improved clinical outcomes. The results contribute to the growing body of evidence supporting radioligand therapy as a viable option for managing mCRPC with visceral metastases.

## Figures and Tables

**Table 1:** Descriptive Statistics of Socio-Demographic Information of Patients.

Parameters	Median (min–max)
Age (year)	72.0 (57–90)
Follow-up periods (months)	6.0 (3–15)
Gleason score	9.0 (6–10)
Lutetium PSMA Cycle Number	4.0 (2–7)
Previous Treatments Received	
Abiraterone	5 (15.6%)
Bicalutamide	19 (59.4%)
Docetaxel	17 (53.1%)
Enza	19 (59.4%)
Goserelin	22 (68.8%)
Leuprolide	17 (53.1%)
Extra liver Metastatic Sites	
Lymph node	1 (3.1%)
Bone	8 (25.0%)
Lymph node + bone	23 (71.9%)
Total	32 (100.0%)
Response rates of liver metastases in control PSMA	
Numerical progress	8 (25.0%)
Molecular progress	5 (15.6%)
Numerical and molecular progress	7 (21.9%)
Numerical regression	1 (3.1%)
Molecular regression	5 (15.6%)
Numerical and molecular regression	6 (18.8%)
Total	32 (100.0%)
ECOG score	
0	5 (15.6%)
1	18 (56.3%)
2	9 (28.1%)
Response to Therapy	
No (Progressive disease)	20 (62.5%)
Yes (Partial regression)	12 (37.5%)
Total	32 (100.0%)
Survival status	
Dead	4 (12.5%)
Alive	28 (87.5%)

ECOG: Eastern Cooperative Oncology Group; PSA: Prostate-Specific Antigen; SUVmax: Maximum Standardized Uptake Value; PFS: Progression-Free Survival; OS: Overall Survival.

**Table 2:** Comparison of pre- and post-treatment parameters.

	Pre-Treatment	Post-Treatment	*p* Value
PSA (ng/mL)	78.0 (5.0–602.0)	70.5 (5.0–699.0)	0.121
Liver SUVmax	16.75 (7.2–42.0)	15.4 (8.3–57.0)	0.242

**Table 3:** Comparison of parameters according to response to therapy in patients receiving Lu-PSMA-617 treatment.

Parameters	No (Progressive) (n = 20)	Yes (Partial Regression) (n = 12)	*p* Value
Age (year)	73.0 (57–90)	71.5 (59–89)	0.697
Follow-up periods (months)	5.0 (3–13)	8.0 (4.0–15.0)	0.009 **
Gleason score	9.0 (6–10)	8.5 (7–10)	0.463
Lutetium PSMA cycle number	3.0 (2–7)	5.0 (3–7)	0.003 **
PSA (ng/mL)	90.0 (22–699)	44.5 (5–563)	0.001 **
Liver SUVmax	17.9 (8.3–57.0)	11.8 (8.7–39.8)	0.008 **
Response rates of liver metastases in control PSMA			<0.001 ***
Numerical progress	8 (40.0%)	0	
Molecular progress	5 (25.0%)	0	
Numerical and molecular progress	7 (35.0%)	0	
Numerical regression	0	1 (8.3%)	
Molecular regression	0	5 (41.7%)	
Numerical and molecular regression	0	6 (50.0%)	
ECOG score			0.050
0	2 (10.0%)	3 (25.0%)	
1	10 (50.0%)	8 (66.7%)	
2	18 (40.0%)	1 (8.3%)	
Survival status			0.103
Dead	4 (20.0%)	0	
Alive	16 (80.0%)	12 (100.0%)	

**: *p* < 0.01; ***: *p* < 0.001.

**Table 4:** Correlation analysis of parameters with Lutetium PSMA cycle number.

	Lutetium PSMA Cycle Number	
	Correlation Coefficient (r)	*p* Value
Gleason score	0.024	0.898
PSA	0.305	0.089
Liver SUVmax	−0.359 *	0.043
Response rates of liver metastases in control PSMA (From Progress to regression)	0.450 **	0.010

* *p* < 0.05; ** *p* < 0.001.

**Table 5:** Response rates of liver metastases (Regression) and linear regression analysis of parameters.

		*p* Value	95.0% CI (Lower–Upper)
Lutetium PSMA cycle number	0.939	0.027	0.860–1.182
Liver SUVmax	0.180	0.037	0.002–0.045
Progression status (Partial regressive)	3.945	<0.001	3.189–4.702

## Data Availability

The original contributions presented in the study are included in the article, further inquiries can be directed to the corresponding author.
